# Effect of Pressing Process on Metabolomics Profiling and Sensory Properties: A Comparative Study of Fu Brick Tea Versus Fu Loose Tea from Identical Raw Dark Tea

**DOI:** 10.3390/foods14173053

**Published:** 2025-08-29

**Authors:** Yan Liang, Jialin Zou, Fanhua Wu, Xiaofang Zhu, Xin Hu, Haoan Zhao, Wei Cao

**Affiliations:** 1 College of Food Science and Technology, Northwest University, Xi’an 710069, China; lianyan5517@126.com (Y.L.); zoujialin@stumail.nwu.edu.cn (J.Z.); wufanhua_1@163.com (F.W.); zhaohaoan@nwu.edu.cn (H.Z.); 2 Key Laboratory of Fu Tea Processing and Utilization, Ministry of Agriculture and Rural Affairs, Xianyang 712044, China; zhuxf819@163.com (X.Z.); huxin@hotmail.com (X.H.); 3 Xianyang Jingwei Fu Tea Co., Ltd., Xianyang 712044, China

**Keywords:** Fu brick tea, Fu loose tea, raw dark tea, metabolomics, gallic acid, catechin

## Abstract

Fu tea is a unique microbially fermented tea from China, and has two types. The primary distinction between these two types—Fu brick tea (FBT) and Fu loose tea (FLT)—is that FBT is compressed into bricks, whereas FLT is maintained as loose leaves. To investigate the differences in the chemical composition and sensory characteristics between the two types of Fu tea, this study utilized samples produced from the same batch of raw dark tea material to ensure comparability. Multiple analytical approaches were applied, including main active component analysis, sensory flavor evaluation, metabolomics, and differential characteristic component analysis. These methods were employed to comprehensively compare and characterize the two tea types. The results showed that compared to FBT, FLT exhibited a longer-lasting fungal flower aroma and a mellower taste. Furthermore, the quantity of *Eurotium cristatum* in FLT was 56.7% higher than that in FBT, indicating a significant difference. Untargeted ultrahigh-performance liquid chromatography–mass spectrometry was used to screen metabolites in Fu tea samples, and after multivariate statistical analysis, 12 differential metabolites were finally identified, including phenolic acids and their derivatives, coumarins and their derivatives, phenylpropanoids, and flavonoids and their glycosides. Subsequently, a targeted UHPLC-QQQ-MS/MS-based method was established and validated for the main differential metabolites, phenolic acids and catechins. The results indicated that gallic acid, catechin, and epicatechin can serve as characteristic markers for distinguishing between FBT and FLT. Notably, the content of gallic acid in FLT was 168.6% higher than that in FBT. These findings elucidate the impact of the pressing process on Fu tea, provide guidance for discriminating between FBT and FLT, and are significant for quality control in the industrial production of Fu tea.

## 1. Introduction

Tea, one of the most widely consumed beverages worldwide, is traditionally made from the leaves of *Camellia sinensis*. It is traditionally classified into green tea, black tea, yellow tea, white tea, oolong tea, and dark tea according to the differences in manufacturing methods and oxidation degree of the tea polyphenols [1,2,3]. Among these teas, Fu tea is a unique kind of microbially fermented dark tea with *Eurotium cristatum* as the dominant fungus, and its biological activity mainly comes from *E. cristatum* and its metabolites [4,5]. It is native to Hunan and Shaanxi provinces in China. Among them, Jingyang County in Shaanxi Province is the most famous, mainly distributed north of the Jing River and in the hinterland of the Guanzhong Plain. It is produced using raw dark tea rather than fresh tea leaves as raw materials [6,7,8,9,10]. Although Fu tea is also rich in compounds such as tea polyphenols and caffeine, its sensory characteristics and health benefits mainly depend on the metabolites and dominant fungi of raw black tea. They can regulate the physiological functions of human metabolism, lower blood sugar and lipid levels, regulate the gastrointestinal tract, and have anti-inflammatory and immune regulation properties. Therefore, the content of *E. cristatum* and its metabolites is an important indicator for evaluating the quality of Fu tea [11].

**Figure 1:**
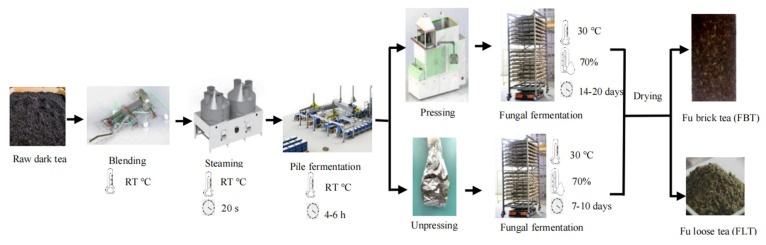
Flow diagram depicting the manufacturing processes used to produce FBT and FLT.

The manufacture of Fu tea mainly consists of the following steps: combined blending, steaming, pile fermentation, pressing or non-pressing, fungal fermentation, and drying [12,13]. Pressing is one of the key processes that lead to the development of the unique characteristics of Fu tea. This process mainly affects the fermentation quality of *E. cristatum.* During pressing, an appropriate moisture content, stem content, and tea brick thickness of the raw tea can provide suitable conditions of water loss and oxygen supply for microbial growth during the fungal fermentation and drying process. Whether the tea is pressed or not distinguishes Fu tea as either Fu brick tea (FBT) or Fu loose tea (FLT) (Figure 1), as it induces significant differences in the fungal fermentation period and flavor qualities between FBT and FLT, especially in terms of appearance, taste, soup color, aroma, and number of *E. cristatum* [12]. The pressing technique has been used for more than 600 years to produce traditional FBT, which is rather well received in the border minority areas for its unique tastes and functions [14,15]. However, FBT needs to be pried apart before drinking, which is not as convenient as FLT, which does not require pressing. In recent years, even though FLT has stricter requirements regarding the processing environment and production techniques, it has been innovatively developed through modern research due to its convenience.

Metabolomics, as a potent tool for analyzing the chemical constituents of plants, has been widely used to elucidate the effects of chemical components and microorganisms on tea and to identify and quantify the metabolites of teas [16,17,18]. Specifically, non-targeted analysis methods can obtain more information on substances, which helps identify differential features between teas [19,20,21]. Rigorous comparisons between distinct teas necessitate strict control over confounding factors. A fundamental strategy to achieve this involves utilizing samples sourced from an identical tea cultivar and from harvests within a narrow temporal window (e.g., consecutive years) [22,23]. Moreover, several previous studies have employed metabolomic approaches to investigate the dynamic changes during the manufacturing process of FBT. For instance, Li comprehensively characterized the metabolic profile and taste evolution throughout the entire production chain of FBT, identifying the key functional microorganisms involved. Their work established a fundamental understanding of how microbial fermentation drives the bioconversion of compounds like catechins, flavonoids, and phenolic acids, leading to a reduction in astringency and bitterness and the development of a mellow taste [24]. Nevertheless, the Fu teas utilized in these studies are predominantly FBT or FLT, but they have seldom been compared. There are few experimental studies on Fu tea products made from the same raw material with different manufacturing processes, which influences the tea quality greatly. The pressing process can alter the firmness and moisture content of raw tea leaves, affecting the water and oxygen supply required by microorganisms for subsequent fungal fermentation. This may result in significantly different fermentation outcomes compared to unpressed tea leaves, ultimately leading to differences in chemical composition. However, detailed information about the effects of “pressing” on the chemical profile of Fu tea is unclear.

In this study, FBT and FLT with the same raw dark tea material were prepared. The main active components and the sensory characteristics were evaluated. Changes in non-volatile metabolites were traced using an ultrahigh-performance liquid chromatography (UHPLC) system coupled to a Q-Exactive Orbitrap tandem mass spectrometer (MS). In addition, the characteristic compounds were identified by UHPLC-QQQ-MS/MS analysis. The expected result was that the pressing step would have a significant impact on the Fu tea, and there would be significant differences in certain components such as *E. cristatum* and catechins between the two types of tea. This analysis aimed to provide new insights into the impact of pressing techniques on the chemical composition of Fu tea in order to improve the processing and quality control of Fu tea and promote its industrial production.

## 2. Materials and Methods

### 2.1. Chemicals and Reagents

Purified water used to brew tea came from Wahaha Group Company (Hangzhou, China). The LC/MS-grade methanol and acetonitrile were from CNW Technologies (Wuppertal, Germany). The formic acid and standards of glucose, theanine, rutin, caffeine, gallic acid, cryptochlorogenic acid, catechin, and epicatechin were obtained from Sigma-Aldrich (St. Louis, MO, USA).

### 2.2. Sample Collection

Briefly, three parallel batches of FBT and FLT samples were obtained from Xianyang Jingwei Fu Tea Co., Ltd. (Xianyang, China), and samples were collected from different production batches. The samples were produced using the same black tea raw materials according to the Chinese Tea Industry Standard GH/T 1246-2019 [25]. Samples were stored at −20 °C before further analysis.

### 2.3. Sensory Evaluation

The method of sensory evaluation refers to previous research [24], and according to the national standard “Methods for Sensory Evaluation of Tea” (GB/T 23776-2018) [26]. We organized a professionally trained sensory panel to conduct sensory evaluation and terminology description of Fu tea samples (5 females, 3 males, aged 30–60 years old). The specific procedure was to take 3 g of tea sample and infuse it in a tea cup with boiling water at 100 °C for 5 min. The tea infusion was then poured immediately into a tea bowl and evaluated by the panelists after brewing. Then, the above steps were repeated to evaluate the tea a total of three times. Finally, the infusion color, taste, fragrance, shape, and the infused tea leaves of the samples were described by the sensory evaluation panel, following the Chinese standard of description that referred to the Tea Vocabulary for Sensory Evaluation [27], and the fragrance and taste were scored mainly based on the second brewing. Each parallel sample of the two types of tea was subjected to three biological replicates, and the result was confirmed when the same comment appeared more than two times.

### 2.4. Determination of Main Active Compounds

The caffeine, total tea polyphenols, and free amino acids were measured according to the spectrophotometry method described in the China standard methods GB/T 8312-2013 [28], GB/T 8313-2018 [29], and GB 8314-2013 [30], respectively. *E. cristatum* was measured according to GB 4789.15-2016 [31]. The soluble sugars and total flavonoids were determined using colorimetric methods based on anthrone and aluminum chloride, the same methods with our previous study [32]. Detailed, step-by-step protocols for the extraction, purification, reaction, and quantification of each component are described in full in the Supplementary Methods file. In brief, the caffeine content was quantified based on its specific UV absorbance at 274 nm. The total tea polyphenol content was determined using the Folin–Ciocalteu method, with gallic acid as a standard, measuring absorbance at 765 nm. The content of free amino acids was assessed via the ninhydrin reaction method, reading absorbance at 570 nm. Soluble sugar content was measured using the anthrone–sulfuric acid method, with glucose as a standard, at 620 nm. The total flavonoid content was determined using a colorimetric assay with rutin as a standard, measuring absorbance at 415 nm. The colony count of Eurotium cristatum was evaluated using the standard plate count method.

### 2.5. Untargeted Metabolomics Analysis

The sample treatments were based on Doppler M.’s method [33] with a few adjustments: 25.0 mg Fu tea sample was extracted using 1 mL extraction solution (methanol–acetonitrile–water = 2:2:1 [*v*/*v*/*v*]) under ultrasound extraction at 40 kHz for 10 min three times; the samples were centrifuged using a TGL-20bR centrifuge (Shanghai, China), produced by Shanghai Anting Scientific Instrument Factory, at 4 °C, 12,000 rpm, 13,800× *g* for 15 min; the supernatant was taken and filtered through a 0.22 μm organic filter membrane. An equal amount of supernatant was mixed to form a quality control (QC) sample for monitoring the stability of the analysis system.

Untargeted metabolomics analysis of non-volatile metabolites was carried out using UHPLC Q-Exactive Orbitrap/MS (Thermo Fisher Scientific, Waltham, MA, USA). Separation of the compounds was conducted using a Phenomenex Kinetex C18 column (2.6 μm, 2.1 mm × 50 mm; Milford, MA, USA) at a flow rate of 0.3 mL/min and temperature of 25 °C. Aqueous formic acid (0.1%, *v*/*v*) and acetonitrile–isopropanol = 1:1 (*v*/*v*) were used as mobile phase A and mobile phase B, respectively. The injection volume was 2 μL. The gradient program was as follows: 0–0.5 min, 1% B; 0.5–4 min, 1–99% B; 4–4.5 min, 99% B; 4.5–4.55 min, 99–1% B; 4.55–6 min, 1% B.

The mass spectrometer was operated at spray voltages of 3.8 kV (+) and 3.4 kV (−); the mass range was m80–1200 *m/z*; the capillary temperature was 320 °C; the heater temperature was 350 °C; the sheath gas pressure was 50 Arb; and the auxiliary air pressure was 15 Arb.

### 2.6. Quantification of Targeted Compounds

A Fu tea sample of 1.000 g (±0.01 g) was homogenized with 50 mL 80% aqueous methanol, and the solution was ultrasonically extracted at 40 kHz for 10 min, centrifuged at 4000× *g* for 10 min, and then the supernatant was taken. In order to fully extract the substances from the sample, the above extraction process was repeated with 50 mL 50% aqueous methanol and 50 mL pure methanol, respectively, followed by combining the three extracts and centrifuging at 12,830× *g* for 10 min. The extracts were stored at −20 °C after mixing with methanol and filtered through 0.22 µm filters before quantitative analysis.

The quantitative analysis of the target compounds in Fu tea samples was performed on an Agilent 1290 series UHPLC system equipped with an Agilent Infinity II 6470 Triple Quad mass spectrometer (Agilent Technologies, Inc., Santa Clara, CA, USA). The chromatographic separation was performed using a constant-temperature (30 °C) Zorbax Eclipse Plus C18 column (50 × 2.1 mm, 1.8 µm; Agilent Technologies, Inc., Santa Clara, CA, USA). The linear gradient elution of the mobile phase which consisted of 0.1% (*v*/*v*) formic acid in water (A) and acetonitrile with 0.1% (*v*/*v*) formic acid (B) with a flow rate of 0.3 mL/min was as follows: 0–1 min, 5% B; 1–5 min, 5–20% B; 5–15 min, 20–24% B; 15–25 min, 24–30% B; 25–30 min, 30–95% B; 30–35 min, 95–5% B; 35–38 min, 5% B with a post-time of 3 min to balance the column. The injection volume was 1 μL.

The optimized MS parameters, operated at ESI ion source voltage of 4.0 kV (+) and 3.5 kV (−) using multiple reaction monitoring (MRM) mode, were as follows: gas temperature, 300 °C; gas flow, 5 L/min; sheath gas temperature, 250 °C; sheath gas flow, 11 L/min; nebulizer pressure, 45 psi. The target compound was quantitatively analyzed using the standard curve obtained from the relationship between the concentration and peak area of the standard sample, mass spectrometry information, and retention time.

### 2.7. Method Validation

Method validation followed the United States Pharmacopeia Convention [34]. The parameters used to determine the reliability of the established UHPLC-QQQ-MS/MS quantitative method for detecting target compounds included linearity and recovery rate. Six concentrations of the target compound were uniformly taken, with the range from 0.50 μg/mL to 100.00 μg/mL as the horizontal axis, and the corresponding peak areas at each concentration as the vertical axis. After fitting, a standard curve was obtained. If R^2^ > 0.99, this indicates a good linear relationship. The calibration curve was obtained based on the relationships between the targeted compound concentrations at six levels ranging from 0.50 μg/mL to 100.00 μg/mL and their corresponding peak areas, which showed good linearity (R^2^ > 0.99). The sensitivities, represented by limits of detection (LOD) and quantification (LOQ), were measured at a signal-to-noise ratio (S/N) of 3 and 10, respectively. The recovery was assessed by standard targeted compound spiking into the Fu tea samples. In addition, the relative standard deviation (RSD) also needed to be calculated. If it was below 10%, this indicated that the experimental data had good technical accuracy and reliability.

### 2.8. Statistical Analysis

Drawing upon the research of Gao [35], our definitive identification methodology is outlined as follows: We utilized Compound Discoverer 3.3 software to conduct retention time correction, peak area extraction, peak categorization, imputation of missing values, peak area normalization, and other pertinent analyses on the raw data [36]. We searched through BiotreeDB (V3.0), HMDB, MONA, METLIN, and our internally developed mass spectrometry database, integrating information such as retention time (RT), MS1, MS2, and others to identify unknown metabolites. Multivariate statistical analysis was conducted using SIMCA 13.0 software (Umetrics AB, Umea, Sweden), with principal component analysis (PCA) and orthogonal partial least squares discriminant analysis (OPLS-DA) performed using relative peak intensities, after standardizing all data for analysis. The R^2^ and Q^2^ values served as indicators for model fitting and predictability, respectively. Ultimately, metabolites with a variable importance in projection (VIP) score greater than 1, a univariate statistical analysis *p*-value less than 0.01, and a fold change (FC) greater than 2 were designated as differential metabolites, with ion flow diagrams generated using Origin 2021 software (OriginLab Co., Northampton, MA, USA). SPSS Statistics 21.0 software (SPSS Inc., Chicago, IL, USA) was used for significant difference analysis of data.

## 3. Results and Discussion

### 3.1. Sensory Evaluation and Main Active Compounds

**Figure 2:**
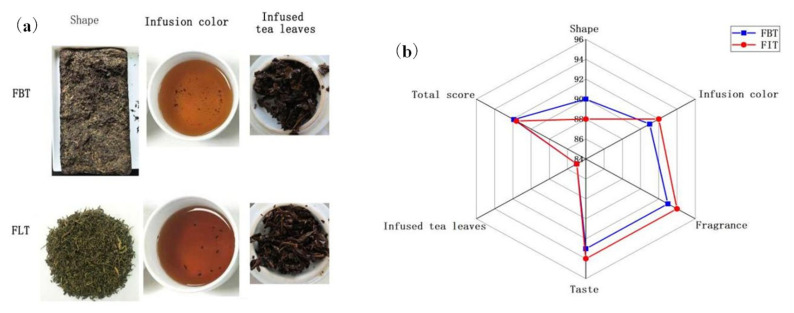
(**a**) The appearance of Fu tea, shape, infusion color, and infused tea leaves of Fu brick tea (FBT) and Fu loose tea (FLT) (from left to right); (**b**) radar plot of sensory descriptor scores of FBT and FLT.

The samples were produced from the same batch of raw dark tea, and according to the sensory evaluation results, both types of tea exhibited the expected sensory characteristics. This also indicated that the production of the two tea samples was successful and that they were of a high quality. Meanwhile, the sensory evaluation results shown in Table 1 and Figure 2a indicated that FLT had a orange red and bright infusion color, more long-lasting “fungal flower” aroma and thicker mellow taste. These sensory differences can be attributed to variations in the chemical and microbial profiles between the two tea types. The stronger fungal aroma in FLT correlates well with its significantly higher content of *E. cristatum*, as shown in Table 2, which likely enhances microbial-derived volatile compounds during fermentation. The mellow taste of FLT may result from increased microbial enzymatic activity, promoting the degradation of polyphenols and polysaccharides, thereby reducing astringency and improving sweetness and thickness. In contrast, the pressing process involved in FBT might restrict microbial proliferation and metabolite diffusion, leading to a comparatively milder aroma and taste. Due to the proportion of each major factor being different between the pressed tea and loose tea [26], the total sensory evaluation score of FBT made from the same raw material is slightly higher (Figure 2b), but with no significant difference.

The main active compositions (Table 2) showed that there was no significant difference (*p* > 0.05) in the content of tea polyphenols, caffeine, free amino acids, total flavonoids, and soluble sugars between FBT and FLT. This lack of divergence suggests that the fundamental chemical profile of the dark tea raw material remains largely unaffected by whether the tea is pressed into a brick or remains loose, highlighting that these particular components may be stable during post-fermentation processing. However, the quantity of *E. cristatum* in FLT was much higher than that in FBT, showing an increase of about 57.6%, with a significant difference between the two types of tea (*p* < 0.05). This pronounced difference may be attributed to the greater oxygenation and more uniform microenvironment available in the loose tea format, which enhances the growth and metabolic activity of the fungus. The higher microbial load likely contributes to the enhanced mellowness perceived in FLT, as *E. cristatum* plays a critical role in the breakdown of polyphenols and polysaccharides, resulting in a smoother and more harmonious flavor profile.

### 3.2. Untargeted Metabolomic Analysis of Non-Volatile Metabolites

#### 3.2.1. Global Metabolite Profiles

**Figure 3:**
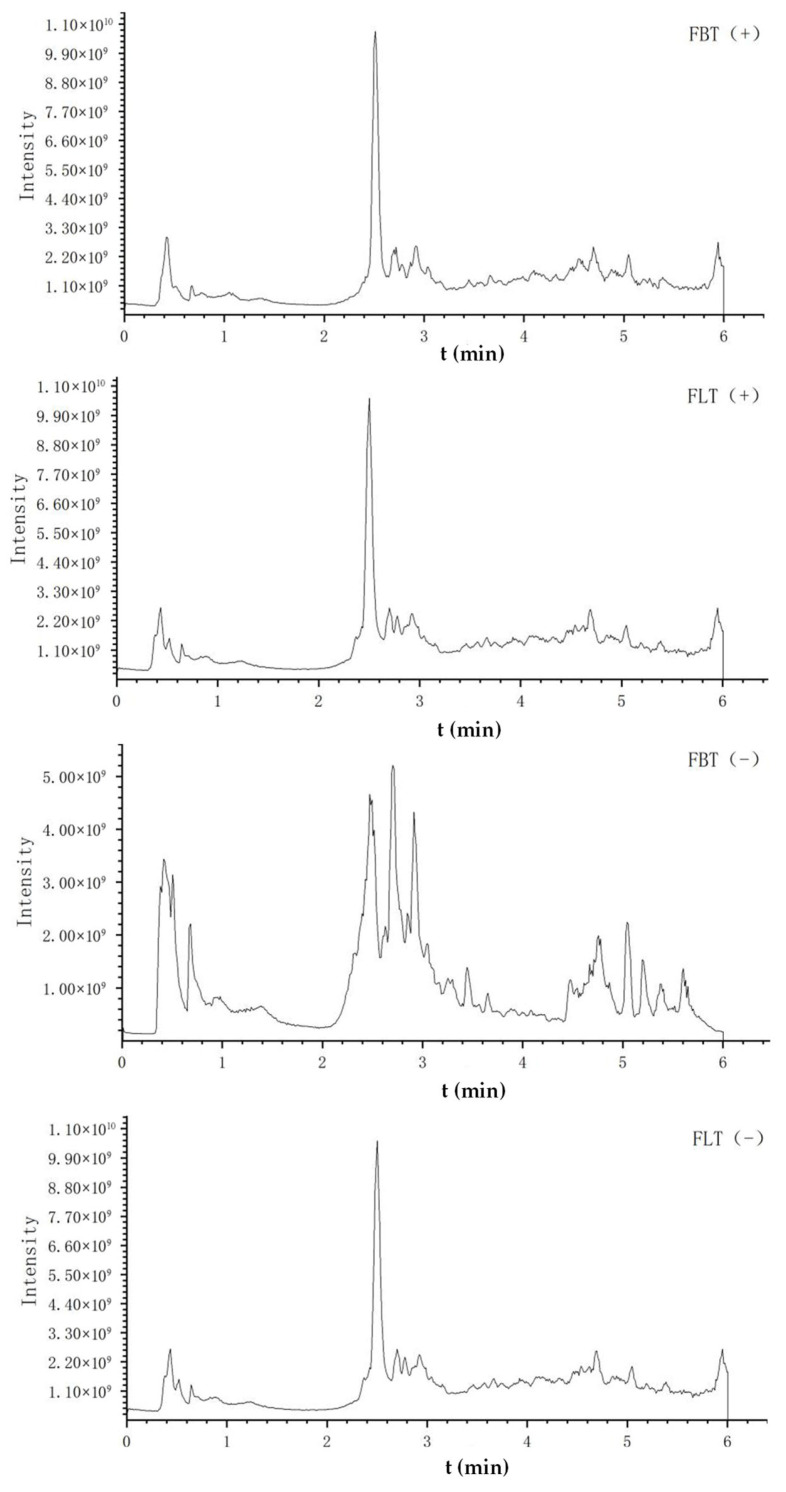
The total ion chromatogram (TIC) of metabolomics analysis of Fu tea samples by UHPLC-Q-Exactive Orbitrap-MS/MS under positive (+) and negative (−) ionization polarity modes.

Figure 3 shows the typical total ion chromatogram (TIC) of FBT and FLT in different modes, respectively. The TICs of FBT and FLT obtained in the positive ion mode were very similar, while those obtained in the negative ion mode before and after 3 min and 4.5 min were significantly different, which was mainly manifested in the peak height and peak area. The comprehensive analysis suggested that the difference between FBT and FLT may be related to compound levels, laying the foundation for subsequent multivariate statistical analysis.

In order to conduct a more comprehensive analysis, we used multidimensional pattern recognition to comprehensively analyze the compounds in the sample, so that we could gain further understanding of the compositional differences between FBT and FLT. We annotated 1386 and 1126 metabolites in the positive and negative ion modes, respectively, based on BiotreeDB (V3.0), HMDB (https://hmdb.ca/, accessed on 7 April 2024), MONA (https://mona.fiehnlab.ucdavis.edu/, accessed on 7 April 2024), METLIN (https://metlin.scripps.edu/, accessed on 7 April 2024), and a self-built mass workstation database, by performing deviation filtering and missing value filtering on the raw data [37]. The complete list of all annotated metabolites is provided in the supplementary dataset in the Supplementary Materials. The confidence of metabolite identification was stratified according to the reporting standards proposed by the Metabolomics Standards Initiative (MSI) [38]. Metabolites identified by comparison with authentic reference standards, matching both retention time and MS/MS fragmentation patterns, were assigned to Level 1 (confirmed structure). The differential metabolites discussed in this study were all identified at this highest confidence level (Level 1).

#### 3.2.2. PCA

**Figure 4:**
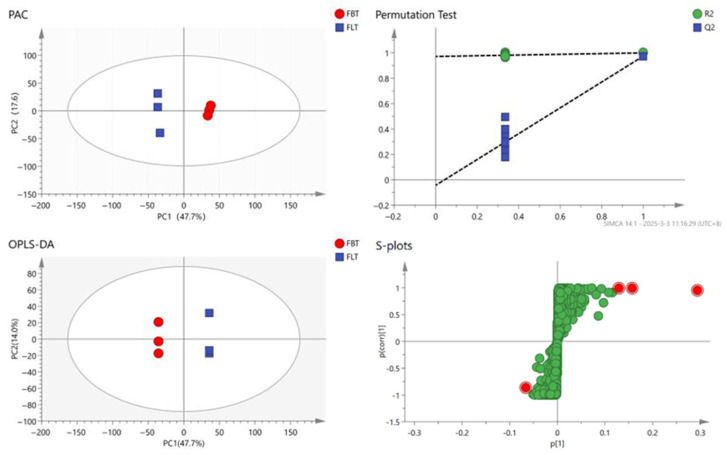
Principal component analysis (PCA) score plot; orthogonal partial least squares discriminant analysis (OPLS-DA) score plot; permutation test plot: The dotted lines represent the R^2^ and Q^2^ values of the original, non-permuted model between FBT and FLT; s-plots for FBT and FLT: The red dots represent the highlighted compounds selected, while the green dots represent the remaining compounds.

To further reveal the metabolic changes that occurred among the FBT and FLT samples, the processed dataset was analyzed using multivariate statistics (Figure 4). A principal component analysis (PCA) was used to reflect the content of metabolites in the sample. The closer the samples are, the more similar they are. In total, 2512 metabolite signals from the Fu tea samples were detected using the UHPLC-Q-Exactive Orbitrap-MS system, and the PCA result (Figure 4) clearly differentiated the FBT and FLT samples, while the contribution rate of PC1 was 47.7%, and the contribution rate of PC2 is 17.6%. The FBT was evenly distributed on the positive half of the horizontal axis, and the FLT was evenly distributed on the negative half of the horizontal axis. The PCA indicated that the repeatability of the Fu tea produced by the same process was good; meanwhile, there were certain differences in the non-volatile metabolites of Fu tea products processed using different methods.

#### 3.2.3. OPLS-DA Analysis

To achieve higher separation levels and more accurate results, the statistical method of the orthogonal partial least squares discriminant analysis (OPLS-DA) model was established to investigate which metabolites could be used for analysis [39]. The Q^2^ value reflects the model’s predictive capability, with proximity to 1 indicating greater stability and reliability. Models exhibiting both R^2^ and Q^2^ values above 0.5 demonstrate acceptable fitting performance [40]. All samples were within the 95% confidence interval and 200 cross-validations were performed on the model. The result (Figure 4) indicated that the predictive ability of this model was effective, and most of the data had been simulated with a cumulative prediction rate of Q^2^ = 0.974, R^2^X = 0.767 and R^2^y = 1. The FBT and FLT samples exhibited a clear separation across the confidence interval, and the discrimination effect was relatively obvious, indicating that there was a significant difference in the composition of the Fu tea samples made with the two different processing techniques; this distinct discrimination supports subsequent differential metabolite identification.

#### 3.2.4. Marker Metabolites

Based on the OPLS-DA results, the variable importance in the projection (VIP) value of each metabolite was analyzed. The higher the VIP value, the greater the contribution of the compound in distinguishing between the two sample groups. To further screen for differential metabolites, 12 differential metabolites were identified between FBT and FLT by using VIP > 2 and *p* < 0.05 (Table 3).

According to the retention time, standard samples, databases, and reference comparisons of various metabolites, the 12 differential metabolites were found, with polyphenolic compounds being the main ones, which comprised important antibacterial and antioxidative constituents in Fu tea [41,42,43]. The critical metabolites were classified as three phenolic acids and their derivatives, four coumarins and their derivatives, one phenylpropanoid, and four flavonoids and their glycosides. These compounds have diverse structures, but all possess strong antioxidant properties, demonstrating synergistic value in preventing metabolic diseases, inflammation, and age-related diseases. The results (Table 3) showed that only epicatechin was identified in the positive ion mode; the rest of the 11 differential metabolites were identified in the negative ion mode. Among them, four metabolites were significantly upregulated and one metabolite was significantly downregulated (when the fold change (FC) ≥ 2, this indicates differential upregulation; when FC ≤ 0.5, it indicates a decrease in the difference) [44].

Among the twelve differential metabolites identified, phenolic acids and catechins were prioritized for further targeted quantification based on the following criteria: (1) Higher VIP values and fold changes: gallic acid, catechin, and epicatechin exhibited among the highest VIP scores and most pronounced fold changes, indicating their paramount importance in discriminating between FBT and FLT. (2) Biological relevance and reported significance: these compounds are well-established as key bioactive constituents in tea, directly associated with its taste (astringency and bitterness) and health benefits (antioxidant activity). (3) Practicality for quality control: gallic acid and catechins are more routinely quantified in tea quality standards, making them practical candidates for developing applicable discrimination methods. Therefore, we focused on gallic acid, cryptochlorogenic acid, catechin, and epicatechin for subsequent validation and quantification to provide a robust and meaningful chemical basis for distinguishing the two tea types.

#### 3.2.5. Quantitative Analysis of Characteristic Components

As the main functional components in Fu tea, metabolites are crucially influenced by processing technique, which contributes to the health benefits of Fu tea. Thus, a UHPLC-Q-TOF-MS/MS-based metabolomic analysis was conducted to screen characteristic components that can discriminate FBT and FLT. Before analyzing the quantity of metabolites in the tea samples, method validation was performed to examine the reliability of the established UHPLC-QQQ-MS/MS method, and the results are shown in Table 4. A certain amount of standard samples of gallic acid, catechin, cryptochlorogenic acid, and epicatechin were diluted into a mixed solution at concentrations of 0.50, 1.00, 5.00, 10.00, 50.00, and 100.00 μg/mL.

A calibration curve was constructed with the standard sample concentration (μg/mL) as the *X* axis and peak area as the *Y* axis. The R^2^ values of fit for gallic acid, catechin, cryptochlorogenic acid, and epicatechin were 0.9941, 0.9916, 0.9945, and 0.9907, respectively, which indicated that the linear equations showed a good linear relationship. The LODs of the four compound quantification methods mentioned above were 0.0067–0.4455 μg/mL, and the LOQ range was 0.0222–1.4850 μg/mL. The RSD (relative standard deviation) values were 0.91%, 3.56%, 3.64%, and 1.87%, respectively. The spiked recovery rates were 97.12%, 97.96%, 95.91%, and 93.43%, suggesting the good reproducibility and accuracy of the method.

**Figure 5:**
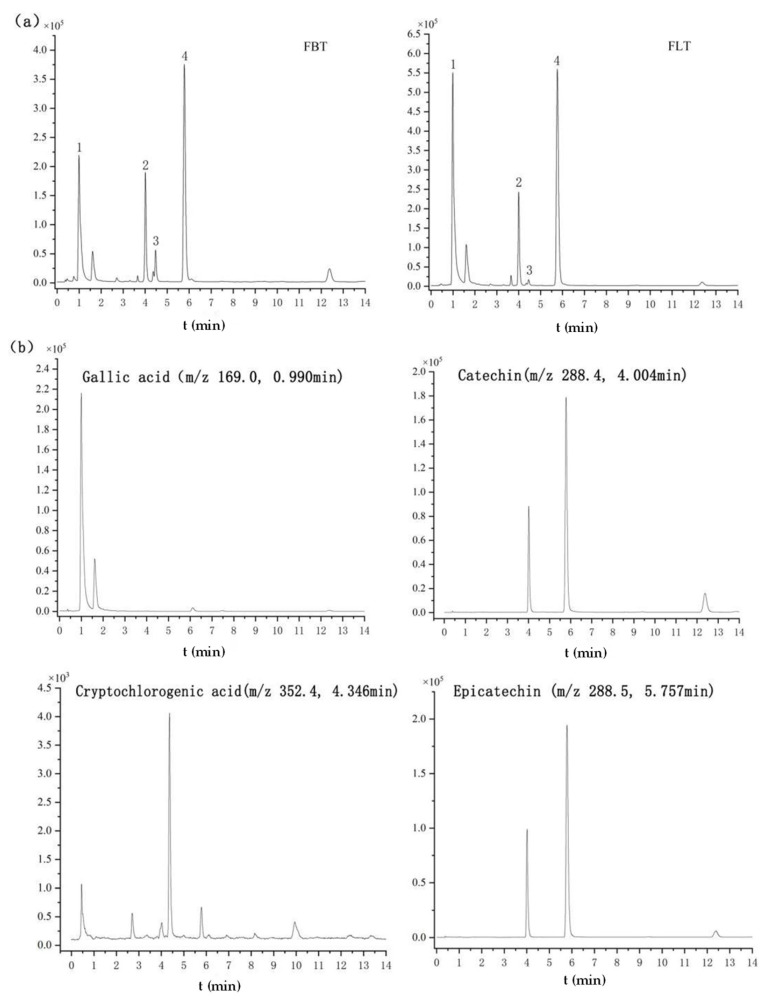
Metabolomics analysis of Fu tea samples by UHPLC-Q-TOF-MS/MS under negative ionization polarity mode. (**a**) The typical total ion chromatogram (TIC) of FBT and FLT (1. gallic acid; 2. catechin; 3. cryptochlorogenic acid; 4. epicatechin). (**b**) Extracted ion chromatograms (EICs) of the four identified compounds.

Based on the established quantitation method using UHPLC-QQQ-MS/MS, the contents of gallic acid, cryptochlorogenic acid, catechin, and epicatechin were evaluated, and the results are summarized in Table 4 and Figure 5. Among them, Figure 5a, b show the TIC of the FBT and FLT and the EIC of four identified compounds, respectively. The significantly higher content of gallic acid (168.6% increase in FLT) is particularly noteworthy. Gallic acid in dark tea is primarily derived from the microbial hydrolysis of galloylated catechins (e.g., EGCG) by fungal tannase [45,46]. The enhanced microbial activity in FLT logically accelerates this bioconversion process. This finding aligns with Yao, who reported that higher fermentation temperatures (which also increase microbial activity) led to an increase in gallic acid content in loose tea [46]. Gallic acid not only contributes to health benefits but also influences taste, potentially mitigating astringency by transforming into less astringent forms than its precursor catechins. The parallel increase in catechin and epicatechin in FLT, although less dramatic, is equally intriguing. It suggests that the pressing process might not only hinder the degradation of catechins but also possibly their de novo synthesis or transformation from other phenolic complexes by microbial enzymes. Alternatively, the compact structure of FBT might physically trap these compounds, making them less extractable. Further investigation is needed to pinpoint the exact mechanism. Nevertheless, the combination of higher levels of these catechins and gallic acid provides a chemical basis for the thicker, mellower taste perceived in FLT, as they contribute to the overall complexity and umami perception of the tea infusion.

## 4. Conclusions

This study established an untargeted metabolomic method based on UHPLC-Q-Exactive Orbitrap-MS technology, which can distinguish between FBT and FLT. The identification and comparison of FBT and FLT showed that this UHPLC-QQQ-MS/MS quantitative method had good repeatability and precision. In conclusion, this study demonstrates that the pressing process critically modulates the microbial ecology and metabolome of Fu tea. The unpressed FLT showed significantly higher *E. cristatum* counts and elevated levels of gallic acid, catechin, and epicatechin, which collectively explain its superior sensory attributes. We attribute these differences to suppression in pressed FBT, which impedes fungal activity and the subsequent bioconversion of polyphenols. This work is significant for the identification of features of FLT and FBT. For industrial applications, it is recommended to use *E. cristatum* counting and gallic acid quantification as key quality indicators, while FLT has become a promising alternative to traditional brick tea due to its convenience and enhanced flavor. This underscores the pressing process not merely as a shaping step but as a crucial regulator of microbial fermentation in Fu tea manufacture. Future work should focus on analyzing the association between characteristic compounds and health benefits, and exploring optimal suppression parameters to maximize microbial efficacy.

## Figures and Tables

**Table 1:** Sensory evaluation results of Fu brick tea (FBT) and Fu loose tea (FLT).

Evaluation Factor	FBT	FLT
Shape	brick-shaped, clean and tight, black auburn, flourishing golden flora	tight and heavy, black auburn, flourishing golden flora
Infusion color	deep yellow and bright	orange red and bright
Fragrance	pure, sharp “fungal flower” aroma	pure, sharp “fungal flower” aroma, comparatively long-lasting
Taste	mellow, fungus flavor	mellow and comparatively thick, fungus flavor

**Table 2:** The main active compositions of Fu brick tea (FBT) and Fu loose tea (FLT).

	Tea Polyphenols (%)	Caffeine (%)	Free Amino Acids (%)	Total Flavonoids (%)	Soluble Sugars (%)	*Eurotium cristatum* (CFU/g)
FBT	11.46 ± 0.20 ^a^	3.26 ± 0.05 ^a^	1.40 ± 0.01 ^a^	2.29 ± 0.15 ^a^	6.43 ± 0.01 ^a^	16.67 ± 0.58 × 10^5 a^
FLT	11.91 ± 0.11 ^a^	3.41 ± 0.06 ^a^	1.31 ± 0.01 ^a^	2.28 ± 0.05 ^a^	6.55 ± 0.08 ^a^	26.0 ± 2.00 × 10^5 b^

Note: Different letters in the same column indicated significant differences (*p* < 0.05). Tool: SPSS Statistics 21.0 software (SPSS Inc., Chicago, IL, USA).

**Table 3:** Critical compounds (VIP > 2.0) responsible for the classification of Fu brick tea (FBT) and Fu loose tea (FLT).

No	Tentative Identification	Rt (min)	Formula	Ion	*m*/*z*	HMDBID	VIP	*p*<	FC (FBT/FLT)	Classification
1	Gallic acid	0.62	C_7_H_6_O_5_	[M-H]^−^	169.0136	HMDB0005807	16.22820	0.01	0.43489	phenolic acid and their derivatives
2	Cichoriin	2.33	C_15_H_16_O_9_	[M-H_2_O-H]^−^	321.0618	HMDB0030821	5.96479	0.05	0.64073	coumarins and derivatives
3	Esculin	2.33	C_15_H_16_O_9_	[M-H_2_O-H]^−^	321.0618	HMDB0030820	5.96479	0.05	0.64073	coumarins and derivatives
4	Cryptochlorogenic acid	2.39	C_16_H_18_O_9_	[M-H]^−^	352.0883	HMDB0030653	3.63898	0.05	2.89865	phenolic acid and their derivatives
5	Scopolin	2.39	C_16_H_18_O_9_	[M-H]^−^	353.0883	HMDB0303366	3.63898	0.05	2.89865	coumarins and derivatives
6	Skimmin	2.46	C_15_H_16_O_8_	[M-OH]^−^	307.0817	HMDB0258334	7.04588	0.01	0.67728	coumarins and derivatives
7	Leucocyanidin	2.48	C_15_H_14_O_7_	[M-H]^−^	305.0668	HMDB0303660	7.59678	0.01	0.76114	flavonoids and their glycosides
8	Corilagin	2.51	C_27_H_22_O_18_	[M-H]^−^	633.0752	HMDB0031457	4.87622	0.01	3.72539	phenolic acid and their derivatives
9	4-*O-p*-Coumaroylquinic acid	2.64	C_16_H_18_O_8_	[M-H]^−^	337.0926	HMDB0302093	7.32125	0.01	2.59944	phenylpropanoids
10	Epicatechin	2.70	C_15_H_14_O_6_	[M-H]^−^	288.0869	HMDB0001871	6.98214	0.01	0.65723	catechins
11	Catechin	2.70	C_15_H_14_O_6_	[M-H]^−^	288.0712	HMDB0002780	8.54179	0.01	0.61807	catechins
12	Myricetin	3.24	C_15_H_10_O_8_	[M-H]^−^	317.0302	HMDB0002755	2.58384	0.05	0.75458	flavonoids and their glycosides

**Table 4:** The contents and method validation parameters of characteristic metabolites of Fu brick tea (FBT) and Fu loose tea (FLT).

Metabolite Name	FBT (mg/g)	FLT (mg/g)	Linear Regressive Equation	R^2^	LOD (μg/mL)	LOQ (μg/mL)	RSD (%)	Recovery Rate (%)
Gallic acid	4.0331	10.8335	Y = 4.1650 × 10^4^ × X + 4.5101 × 10^5^	0.9941	0.3116	1.0387	0.91	97.12
Catechin	0.4982	0.7418	Y = 3.1856 × 10^5^ × X + 2.9830 × 10^5^	0.9916	0.4455	1.4850	3.56	97.96
Cryptochlorogenic acid	1.1350	1.0725	Y = 3.0857 × 10^4^ × X − 2.1402 × 10^5^	0.9945	0.0067	0.0222	3.64	95.91
Epicatechin	4.0772	5.5228	Y = 5.1177 × 10^4^ × X − 5.6633 × 10^4^	0.9907	0.2266	0.7553	1.87	93.43

## Data Availability

The original contributions presented in this study are included in the Supplementary Materials. Further inquiries can be directed to the corresponding authors.

## Supplementary Materials

The following supporting information can be downloaded at https://www.mdpi.com/article/10.3390/foods14173053/s1: Specific methods for measuring caffeine, total tea polyphenols, free amino acids, *E. cristatum*, soluble sugars and total flavonoids; Table S1: Metabolite raw data.

## References

[1] Wang H., Provan G.J., Helliwell K. (2000). Tea flavonoids: Their functions, utilisation and analysis. Trends Food Sci. Technol..

[2] Luo Z.M., Ling T.J., Li L.X., Zhang Z.Z., Zhu H.T., Zhang Y.J., Wan X.C. (2012). A new norisoprenoid and other compounds from fuzhuan Brick tea. Molecules.

[3] Wang M., Chen G., Chen D., Ye H., Liu Z. (2019). Purified fraction of polysaccharides from fuzhuan brick tea modulates the composition and metabolism of gut microbiota in anaerobic fermentation in vitro. Int. J. Biol. Macromol..

[4] Xu A., Wang Y., Wen J., Liu P., Liu Z., Li Z. (2011). Fungal community associated with fermentation and storage of Fuzhuan brick-tea. Int. J. Food Microbiol..

[5] Tan Z.W., Yu P.L., Zhu H.Y., Gao J.B., Han N., Yang C.C., Shen Z., Gao C., Yang X.B. (2024). Differential characteristics of chemical composition, fermentation metabolites and antioxidant effects of polysaccharides from *Eurotium cristatum* and Fu-brick tea. Food Chem..

[6] Mo H., Zhu Y., Chen Z. (2008). Microbial fermented tea–a potential source of natural food preservatives. Trends Food Sci. Technol..

[7] Lv H.P., Zhang Y., Shi J., Lin Z. (2017). Phytochemical profiles and antioxidant activities of Chinese dark teas obtained by different processing technologies. Food Res. Int..

[8] Zhang X., Chen H., Zhang N., Chen S., Tian J., Zhang Y., Wang Z. (2013). Extrusion treatment for improved physicochemical and antioxidant properties of high-molecular weight polysaccharides isolated from coarse tea. Food Res. Int..

[9] Du Y., Yang W.R., Yang C.C., Yang X.B. (2022). A comprehensive review on microbiome, aromas and flavors, chemical composition, nutrition and future prospects of Fuzhuan brick tea. Trends Food Sci. Technol..

[10] Zheng W.J., Wan X.C., Bao G.H. (2015). Brick dark tea: A review of the manufacture, chemical constituents and bioconversion of the major chemical components during fermentation. Phytochem. Rev..

[11] Li T.T., Liu Z.J., Liu S.Q., Li J., Zheng Y.J., Liu Z.H., Ling P.X. (2023). High-Value Utilization of Tea Forest Resources: Breeding *Eurotium cristatum* Strains to Enhance Lovastatin Yields in Anhua Dark Tea. Forests.

[12] (2018). Black Tea-Part 5: Fu Tea.

[13] Li Q., Li Y., Luo Y., Xiao L., Wang K., Huang J., Liu Z. (2020). Characterization of the key aroma compounds and microorganisms during the manufacturing process of Fu brick tea. Lebensm.-Wiss.-Technol..

[14] Wang Y., Xu A., Liu P., Li Z. (2015). Effects of Fuzhuan Brick-tea water extract on mice infected with *E. coli* O157:H7. Nutrients.

[15] Chen G., Xie M., Wan P., Chen D., Ye H., Chen L., Zeng X., Liu Z. (2018). Digestion under saliva, simulated gastric and small intestinal conditions and fermentation in vitro by human intestinal microbiota of polysaccharides from Fuzhuan brick tea. Food Chem..

[16] Xiao Y., He C., Chen Y.L., Ho C.Y., Wu X., Huang Y.X., Gao Y., Hou A.X., Li Z.J., Wang Y.L. (2022). UPLC–QQQ–MS/MS-based widely targeted metabolomic analysis reveals the effect of solid-state fermentation with *Eurotium cristatum* on the dynamic changes in the metabolite profile of dark tea. Food Chem..

[17] Long P., Wen M., Granato D., Zhou J., Wu Y., Hou Y., Zhang L. (2020). Untargeted and targeted metabolomics reveal the chemical characteristic of Pu-erh tea (*Camellia assamica*) during pile-fermentation. Food Chem..

[18] Lv C., Chen C., Ge F., Liu D., Zhao S., Chen D. (2013). A preliminary metagenomic study of Pu-erh tea during pile fermentation. J. Sci. Food Agric..

[19] Li Y.Y., Zhou H., Tian T., Hou Y.H., Chen D., Zhou J., Liu S.Y., Yu Y.B., Dai W.D., Zhou T.S. (2023). Nontargeted and targeted metabolomics analysis for evaluating the effect of “golden flora” amount on the sensory quality, metabolites, and the alpha-amylase and lipase inhibitory activities of Fu brick tea. Food Chem..

[20] Shen S.S., Huang J.L., Li T.H., Wei Y.M., Xu S.S., Wang Y.J., Ning J.J. (2022). Untargeted and targeted metabolomics reveals potential marker compounds of an tea during storage. LWT-Food Sci. Technol..

[21] Yue W., Sun W., Rao R.S.P., Ye N., Yang Z., Chen M. (2019). Non-targeted metabolomics reveals distinct chemical compositions among different grades of Bai Mudan white tea. Food Chem..

[22] Wang Y., Kan Z., Thompson H.J., Ling T., Ho C.T., Li D., Wan X. (2019). Impact of Six Typical Processing Methods on the Chemical Composition of Tea Leaves Using a Single *Camellia sinensis* Cultivar, Longjing 43. J. Agric. Food Chem..

[23] Feng Z., Li Y., Li M., Wang Y., Zhang L., Wan X., Yang X. (2019). Tea aroma formation from six model manufacturing processes. Food Chem..

[24] Li Q., Jin Y., Jiang R., Xu Y., Zhang Y., Luo Y., Liu Z.H. (2021). Dynamic changes in the metabolite profile and taste characteristics of Fu brick tea during the manufacturing process. Food Chem..

[25] (2018). Technology Regulations for Fu Tea Processing.

[26] (2018). Methodology for Sensory Evaluation of Tea.

[27] (2017). Tea Vocabulary for Sensory Evaluation.

[28] (2013). Tea-Determination of Caffeine Content.

[29] (2018). Determination of Total Polyphenols and Catechins Content in Tea.

[30] (2013). Tea-Determination of Free Amino Acids Content.

[31] (2016). Microbiological Examination of Food-Method for Counting Fungi and Yeasts.

[32] Liang Y., Wu F., Wu D., Zhu X., Gao X., Hu X., Xu F., Ma T., Zhao H., Cao W. (2024). Fu Loose Tea Administration Ameliorates Obesity in High-Fat Diet-Fed C57BL/6J Mice: A Comparison with Fu Brick Tea and Orlistat. Foods.

[33] Doppler M., Kluger B., Bueschl C., Schneider C., Schuhmacher R. (2016). Stable Isotope-Assisted Evaluation of Different Extraction Solvents for Untargeted Metabolomics of Plants. Int. J. Mol. Sci..

[34] Brittain H.G. (2002). Profiles of drug substances, excipients, and related methodology. Analytical Profiles of Drug Substances and Excipients.

[35] Gao S., Jennings E.K., Han L., Koch B.P., Herzsprung P., Lechtenfeld O.J. (2024). Detection and Exclusion of False-Positive Molecular Formula Assignments via Mass Error Distributions in UHR Mass Spectra of Natural Organic Matter. Anal. Chem..

[36] Kind T., Wohlgemuth G., Lee D.Y., Lu Y., Palazoglu M., Shahbaz S., Fiehn O. (2009). FiehnLib: Mass spectral and retention index libraries for metabolomics based on quadrupole and time-of-flight gas chromatography/mass spectrometry. Anal. Chem..

[37] Zhou Z., Luo M., Zhang H., Yin Y., Cai Y., Zhu Z.J. (2022). Metabolite annotation from knowns to unknowns through knowledge-guided multi-layer metabolic networking. Nat. Commun..

[38] Sumner L.W., Amberg A., Barrett D., Beale M.H., Beger R., Daykin C.A., Fan T.W.M., Fiehn O., Goodacre R., Griffin J.L. (2007). Proposed minimum reporting standards for chemical analysis. Metabolomics.

[39] Trygg J., Wold S. (2010). Orthogonal projections to latent structures (O-PLS). J. Chemom..

[40] Yun J., Cui C.J., Zhang S.H., Hou R. (2021). Use of headspace GC/MScombined with chemometric analysis to identify the geographic origins of black tea. Food Chem..

[41] Zhang Q., Shi Y., Ma L., Yi X., Ruan J. (2014). Metabolomic analysis using ultra-performance liquid chromatography-quadrupole-time of flight mass spectrometry (UPLC-Q-TOF MS) uncovers the effects of light intensity and temperature under shading treatments on the metabolites in tea. PLoS ONE.

[42] Plaza M., Pozzo T., Liu J.Y., Kazi Zubaida G.A., Charlotta T., Nordberg Karlsson E. (2014). Substituent effects on in vitro antioxidizing properties, stability, and solubility in flavonoids. J. Agric. Food Chem..

[43] Lu Y., Jiang F., Jiang H., Wu K.L., Zheng X.G., Cai Y.Z., Katakowski M., Chopp M., To S.T. (2010). Gallic acid suppresses cell viability, proliferation, invasion and angiogenesis in human glioma cells. Eur. J. Pharmacol..

[44] Zhang H.F., Lu Q., Liu R. (2022). Widely targeted metabolomicsanalysis reveals the effect of fermentation on the chemicalcomposition of bee pollen. Food Chem..

[45] Ouyang M., Xiong C., Tu Y.Y., Cheng L., Shu H., Wen T. (2011). Effects of *Eurotium cristatum* on tea quality and antioxidant activity. Mygosystema.

[46] Yao Y.N., Wu M.Y., Huang Y.J., Li C.K., Pan X., Zhu W., Huang Y.Y. (2017). Appropriately raising fermentation temperature beneficial to the increase of antioxidant activity and gallic acid content in *Eurotium cristatum*-fermented loose tea. LWT-Food Sci. Technol..

