# Supplementary material for: Effect of Pressing Process on Metabolomics Profiling and Sensory Properties: A Comparative Study of Fu Brick Tea Versus Fu Loose Tea from Identical Raw Dark Tea
Source: Foods. 2025 Aug 29;14(17):3053. doi: 10.3390/foods14173053 (PMC12428677; doi:10.3390/foods14173053)
Supplement: Supplementary file 1 [file foods-14-03053-s001.zip › Detailed Protocols for Chemical Component Analysis.pdf]

Effect of Pressing Process on Metabolomics Profiling and Sensory Properties: A Comparative Study of Fu Brick Tea *versus* Fu Loose Tea from Identical Raw Dark Tea

Yan Liang [1,2], Jialin Zou [1], Fanhua Wu [1], Xiaofang Zhu [2,3], Xin Hu [2,3], Haoan Zhao [1] and Wei Cao [1,*]

[1] College of Food Science and Technology, Northwest University, Xi'an 710069, China; lianyan5517@126.com (Y.L.); zoujialin@stumail.nwu.edu.cn (J. Z.); wufanhua_1@163.com(F.W.); zhaohaoan@nwu.edu.cn (H.Z.)
[2] Key Laboratory of Fu Tea Processing and Utilization, Ministry of Agriculture and Rural Affairs, Xianyang 712044, China; zhuxf819@163.com (X.Z.); huxin@hotmail.com (X.H.)
[3] Xianyang Jingwei Fu Tea Co., Ltd., Xianyang 712044, China;
* Correspondence: caowei@nwu.edu.cn (W.C.)

Detailed explanation of the method in section 2.4:

Caffeine：

Principle: Caffeine is extracted from tea with boiling water, purified by precipitation (using basic lead acetate and sulfuric acid), and quantified by UV absorbance at 274 nm.

Key Reagents: Basic lead acetate solution (50 g/100 mL), 0.01 mol/L HCl, 4.5 mol/L H_2SO_4, caffeine standard solution (0.05 mg/mL).

Procedure:Extract 3 g of ground tea in 450 mL boiling water (45 min).

Purify extract: Dilute 10 mL extract to 100 mL with 4 mL HCl + 1 mL lead acetate → filter. Add 0.1 mL H_2SO_4 to 25 mL filtrate → dilute to 50 mL → filter.

Measure absorbance at 274 nm vs. reagent blank.

Calculation:

Caffeine(%) =$\frac{C\times V/1000\times 100/10\times 50/25}{m\times\omega}\times 100\%$

C: Caffeine conc. from std curve (mg/mL); V: Total extract vol (mL); m: Sample mass (g); ω: Dry matter content (%).

Free Amino Acids:**Principle:** Amino acids react with ninhydrin at pH 8.0 to form purple complexes, measured at 570 nm.

Key Reagents: pH 8.0 phosphate buffer, 2% ninhydrin solution (with $SnCl_2$), theanine/glutamic acid standards.

Procedure: Prepare tea extract (as in caffeine method).

Mix 1 mL extract + 0.5 mL buffer + 0.5 mL ninhydrin → heat 15 min (100° C) → dilute to 25 mL.

Measure absorbance at 570 nm after 10 min.

Calculation:

Free Amino Acids(%) =$\frac{C\times V/1000\times V_1/V_2}{m\times\omega}\times 100\%$

C: Amino acid from std curve (mg); V_1 : Total extract vol (mL); V_2 : Test aliquot vol (mL); m: Sample mass (g); ω: Dry matter content (%).

Total Tea Polyphenols:

Principle: Polyphenols oxidize Folin-Ciocalteu reagent, forming blue complexes measured at 765 nm. Gallic acid is the calibration standard.

Key Reagents: 70% methanol, 10% Folin-Ciocalteu reagent, 7.5% Na_2CO_3, gallic acid standards.

Procedure: Transfer 1.0mL of gallic acid working solution, water (as blank control), and test solution into graduated test tubes using pipettes.

Mix 1 mL diluted extract + 5 mL Folin reagent + 4 mL Na_2CO_3 → dilute to 10 mL → incubate 60 min (RT).

Measure absorbance at 765 nm.

Calculation:

Tea Polyphenols(%) =$\frac{(A-A_0)\times V\times d\times 100}{SLOPE_{Std}\times m\times\omega\times 10^6}\times 100\%$

A - absorbance of sample testing solution; A_0 -- absorbance of reagent blank solution; $SLOPE_{Std}$ - Slope of the standard curve for gallic acid; m - sample mass, in grams (g); V - volume of sample extraction solution, in milliliters (mL); d - Dilution factor (usually 1 mL diluted to 100 mL, then its dilution factor is 100); ω - Sample dry matter content (%).

Total Flavonoids:
Principle: Flavonoids complex with $AlCl_3$ in pH 5.5 buffer, measured at 415 nm. Rutin is the standard.
Key Reagents: 1.5% $AlCl_3$, pH 5.5 acetate buffer, 50% ethanol, rutin standard.
Procedure: Extract 0.1 g tea in 7 mL 50% ethanol (80°C, 1 h, ultrasonic 10 min) → dilute to 10 mL.
Mix 1 mL extract + 8 mL $AlCl_3$ + 4 mL buffer → dilute to 25 mL → incubate 30 min.
Measure absorbance at 415 nm.
Calculation:

Total Flavonoids(%) $= \frac{C \times 10 \times 25}{m \times \omega \times 1000} \times 100\%$

C: Flavonoid conc. from std curve (mg/mL); m: Sample mass (g); ω: Dry matter content (%)

Soluble Sugars:
Principle: Sugars react with anthrone in concentrated H_2SO_4 to form green complexes, measured at 620 nm. Glucose is the standard.
Key Reagents: Anthrone reagent (0.6 g in 100 mL H_2SO_4 + 33 mL H_2O), glucose standards.
Procedure: Extract 1 g tea in 80 mL boiling water (30 min) → dilute to 500 mL.
Mix 1 mL extract + 8 mL anthrone reagent → heat 7 min (100°C) → cool in ice bath.
Measure absorbance at 620 nm.
Calculation:

Soluble Sugars(%) $= \frac{C/1000 \times L/1000}{m \times \omega} \times 100\%$

C: Sugar conc. from std curve (mg/L); V: Total extract vol (mL); m: Sample mass (g); ω: Dry matter content (%)

***E. Cristatum*:**
Principle: The sample is appropriately diluted and inoculated onto a selective medium (Rose Bengal Agar or Potato Dextrose Agar) containing antibiotics. After incubation at a specified temperature, typical mold colonies are counted. The result is reported as the number of mold colonies per gram (or per milliliter) of sample.
Key Reagents:
Rose Bengal Agar (RBA) or Potato Dextrose Agar (PDA)
Chloramphenicol solution (0.1 g/L)
Sterile saline (0.85% NaCl)
Ethanol (75%), for surface disinfection

Procedure:

Sample Preparation: Aseptically weigh 25 g of sample into 225 mL of sterile saline and homogenize to create a 1:10 dilution.

Serial Dilution: Prepare a series of decimal dilutions (e.g., 10^{-2}, 10^{-3}) in sterile saline as needed.

Inoculation and Incubation:

Transfer 1 mL of each dilution into a sterile petri dish.

Pour approximately 15 - 20 mL of molten Rose Bengal Agar (cooled to 45 - 50°C) into each plate. Swirl gently to mix.

After the agar has solidified, invert the plates and incubate at 28 ± 1°C for 5 to 7 days.

Enumeration: After incubation, select plates containing between 10 and 150 typical mold colonies for counting. Record the number of colonies.

Calculation:

E. Cristatum(CFU/g) = $\frac{N \times D}{m}$

N: Average number of colonies per plate; D: Dilution factor; m: Mass of the test portion (g).
